# Supplementary material for: Comparative Genomics of Apomictic Root-Knot Nematodes: Hybridization, Ploidy, and Dynamic Genome Change
Source: Genome Biol Evol. 2017 Sep 25;9(10):2844–61. doi: 10.1093/gbe/evx201 (PMC5737495; doi:10.1093/gbe/evx201)
Supplement: Supplementary Data [file evx201_supp.pdf]

Comparative genomics of apomictic root-knot nematodes: hybridization, ploidy, and dynamic genome change

Amir Szitenberg[1,2,§], Laura Salazar-Jaramillo[3], Vivian C. Blok[4], Dominik R. Laetsch[3,4], Soumi Joseph[5], Valerie M. Williamson[6], Mark L. Blaxter[3], David H. Lunt[1]

1 Evolutionary Biology Group, School of Environmental Sciences, University of Hull, Kingston upon Hull, UK
2 Microbial Metagenomics Division, Dead Sea and Arava Science Center, Mt. Masada, Israel
3 Institute of Evolutionary Biology, School of Biological Sciences, University of Edinburgh, Edinburgh, UK
4 The James Hutton Institute, Invergowrie, Dundee, UK
5 Department of Entomology and Nematology, University of Florida, Gainesville, USA
6 Department of Plant Pathology, University of California, Davis, USA

§ Correspondence: Amir Szitenberg, amir@adssc.org

Supplementary Material

0 Species sample metadata

Table S1. ***Meloidogyne*** **isolates sequenced in this study.** One draft *M. floridensis* genome was published previously (Lunt et al. 2014a). Some isolates have been in culture for a long time and exact origins are not available.

	Species	Isolate	Sourced	Geographic origin	Notes
1	M. incognita	A14	Vivian Blok	Libya	Adam 2006 (PhD)
2	M. incognita	L27	Vivian Blok	USA	Race 1, Blok et al 1997
3	M. incognita	VW6	Valerie Williamson	California, USA	Isolated from cotton; (Wang et al. 2010)
4	M. incognita	W1	Valerie Williamson	California, USA	Isolated from tomato with the nematode resistance gene Mi-1; (Gross and Williamson 2011)
5	M. incognita	HarC	Valerie Williamson	California, USA	Isolated from the nematode resistant grape variety Harmony; (Ferris, Zheng, and Walker 2012)
6	M. incognita	557R	Valerie Williamson	North Carolina, USA	Isolated from tomato with the nematode resistance gene Mi-1; (Yaghoobi et al. 1995)
7	M. incognita	L19	Vivian Blok	French West Indies	Blok et al 1997
8	M. incognita	L9	Vivian Blok	Ivory coast, Africa	Blok et al 1997
9	M. javanica	VW5	Valerie Williamson	California, USA	Selected from strain VW4 following reproduction on Mi-1

					tomato;<br>(Gleason, Liu, and Williamson 2008)
10	M. javanica	VW4	Valerie Williamson	California, USA	Also PacBio genome; (Yaghoobi et al. 1995)
11	M. javanica	L57	Vivian Blok	Morocco	(Adam, Phillips, and Blok 2005)
12	M. javanica	VB15	Vivian Blok	Unknown	
13	M. javanica	VB17	Vivian Blok	Unknown	
14	M. arenaria	L28	Vivian Blok	French West Indies	Blok et al 1997
15	M. arenaria	L32	Vivian Blok	French West Indies	Blok et al 1997
16	M. arenaria	HarA	Valerie Williamson	California, USA	Isolated from the nematode resistant grape variety Harmony; (Ferris, Zheng, and Walker 2012)
17	M. enterolobii	L30	Vivian Blok	Burkino Faso	Blok et al 1997
18	M. floridensis	SJF1	Soumi Joseph	Florida, USA	Isolated from peach
19	M. floridensis	JB5	Janete Brito	Florida, USA	(Lunt et al. 2014a)
20	M. haplanaria	SJH1	Soumi Joseph	Florida, USA	Isolated from tomato with the nematode resistance gene Mi-1; (Joseph et al. 2016)

Table S2 - Genome assembly statistics

Species	Strain	Insert size	Reads	Size (bp)	Exp. coverage
M. javanica	VW4	300	62,075,861	7,635,287,416	100x
M. javanica	VW4	500	123,728,247	15,168,259,765	200x
M. javanica	VW5	350	193,072,088	24,134,011,000	320x
M. javanica	L57	350	32,669,417	4,083,677,125	27x
M. javanica	L15	350	29,324,182	3,665,522,750	24x
M. javanica	L17	350	31,332,441	3,916,555,125	26x
M. incognita	W1	350	38,260,145	4,782,518,125	63x
M. incognita	W1	550	30,290,198	3,786,274,750	50x
M. incognita	VW6	350	28,840,610	3,605,076,250	48x
M. incognita	VW6	550	25,746,808	3,218,351,000	42x
M. incognita	HarC	350	26,844,521	3,355,565,125	44x
M. incognita	HarC	550	35,340,761	4,417,595,125	58x
M. incognita	557R	550	62,745,198	7,843,149,750	104x
M. incognita	L9	350	19,009,603	2,376,200,375	18x
M. incognita	L19	350	33,486,356	4,185,794,500	28x
M. incognita	L27	350	35,218,809	4,402,351,125	29x
M. incognita	A14	350	20,025,193	2,503,149,125	17x
M. arenaria	HarA	350	49,813,878	6,226,734,750	41x

M. arenaria	HarA	550	46,643,017	9,656,831,750	64x
M. arenaria	L28	350	16,744,391	2,093,048,875	14x
M. arenaria	L32	350	14,159,397	176,9924,625	11x
M. enterolobii	L30	350	143,672,079	17,959,009,875	120x
M. enterolobii	L30	550	100,032,455	12,504,056,875	83x
M. floridensis	SJF1	350	105,579,171	13,197,396,375	175x
M. floridensis	SJF1	550	95,211,681	11,901,460,125	160x

1 Randomization tests for the phylogenetic congruence between genome A and genome B

1.1 Coalescent gene tree based analysis

If the two homoeologues were acquired at the base of MIG as a result of a single hybridization event, then their phylogenetic trees should be congruent and reflect their coevolution. If this is correct, in the reconstruction of a coalescence tree based on 533 gene trees, we may randomly assign the homoeologue annotation to either of the subtree in each of the gene trees (Figure S3A). In each gene tree, homoeologs are denoted 1 and 2 instead of A and B because for most gene pairs we lack synteny information. In Figure S3B, the non-randomized coalescence tree supports the phylogenetic relationships recovered in the maximum likelihood tree (Figure 3). In the non-randomized tree, in each of the gene trees we denoted the slower evolving homoeologue as homoeologue 1. As this is an artificial decision, we can only be confident in the topology of our multi-loci trees, and not in the branch length, as long as there is phylogenetic congruence between the two homoeologues. Figure S3C is the strict consensus of all the randomised coalescence analyses, which also supports all the interspecies relationships within both homoeologue subtrees.

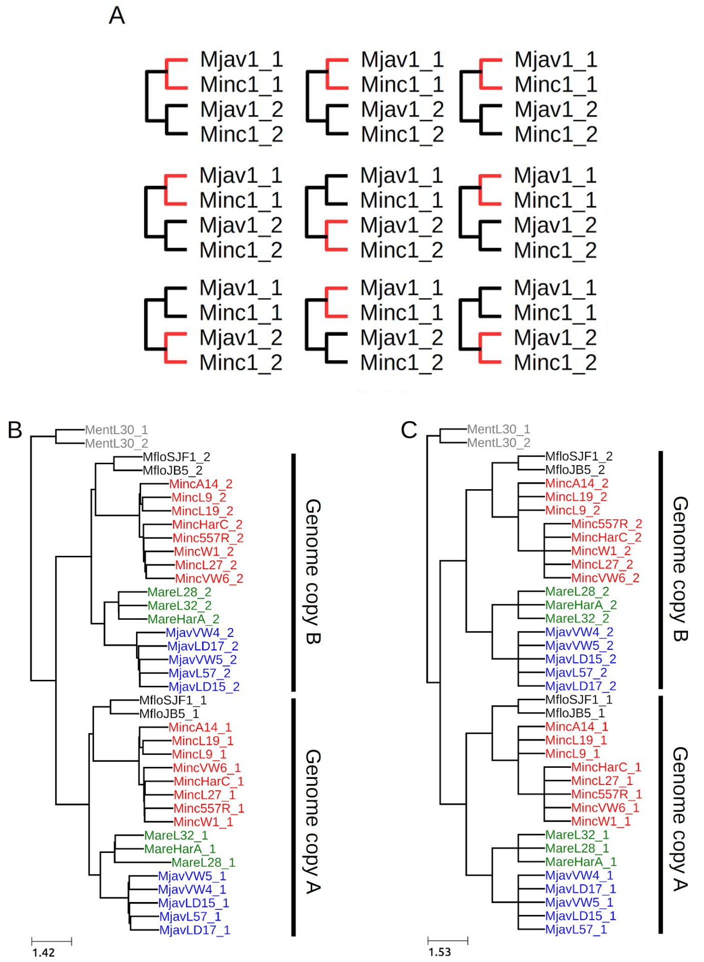

Figure S1: The randomized homoeologue coalescence approach used to confirm the phylogenetic congruence between homoeologue A and B (A), the non-randomised coalescence tree with all the gene trees “pre-ordered” (B), and the resulting strict consensus tree off all the randomized coalescence trees.

1.2 Supermatrix maximum likelihood based analysis

If the two homoeologues were acquired at the base of MIG as a result of a single hybridization event, then their phylogenetic trees should be congruent and reflect their coevolution. If this is correct, we may concatenate the homoeologue A sequence from gene x with homoeologue B sequences from gene y, and *vice versa*, without altering the phylogenetic relationships within each of the homoeologue subtrees, as they are the same (Figure S3A). We produced 100 supermatrices with the 533 nuclear genes used for Figure 3, and randomized the concatenation of the two homoeologues as described above and in Figure S4A. We produced a strict consensus of the resulting 100 trees (Figure S3B), showing that the relationship between species within each of the homoeologue subtrees were recovered in all the 100 trees. This confirms that the two homoeologues share the same phylogenetic history. (See next page for figure legend)

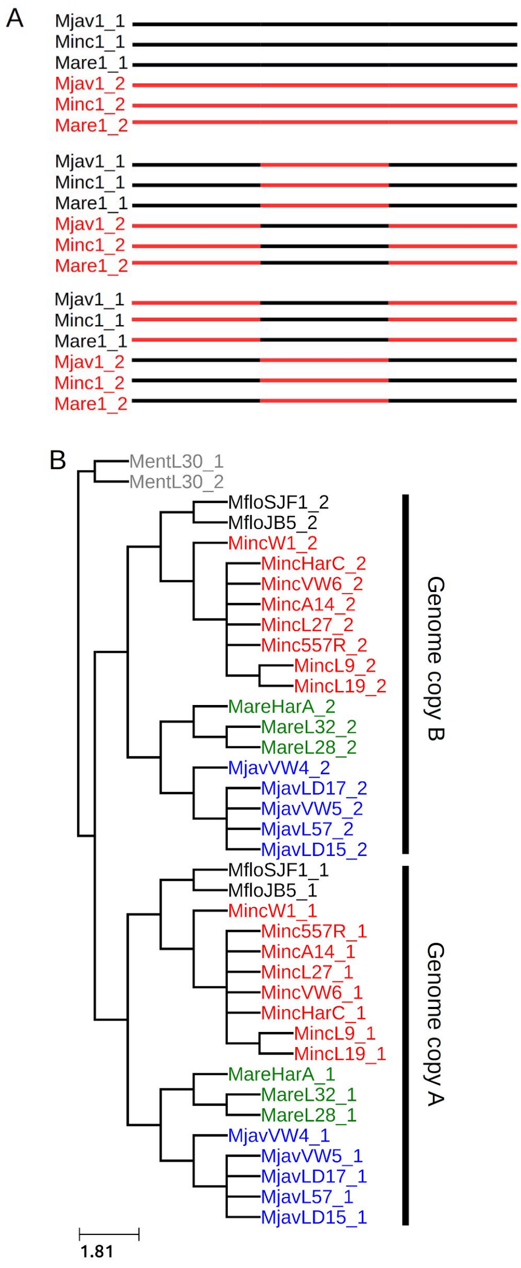

Figure S2: The randomized homoeologue concatenation approach used to confirm the phylogenetic congruence between homoeologue A and B (A), and the resulting strict

consensus tree off all the randomized matrices (B). The homoeologs are denoted 1 and 2 instead of A and B because for most gene pairs we lack synteny information.

2. Gene conversion

A

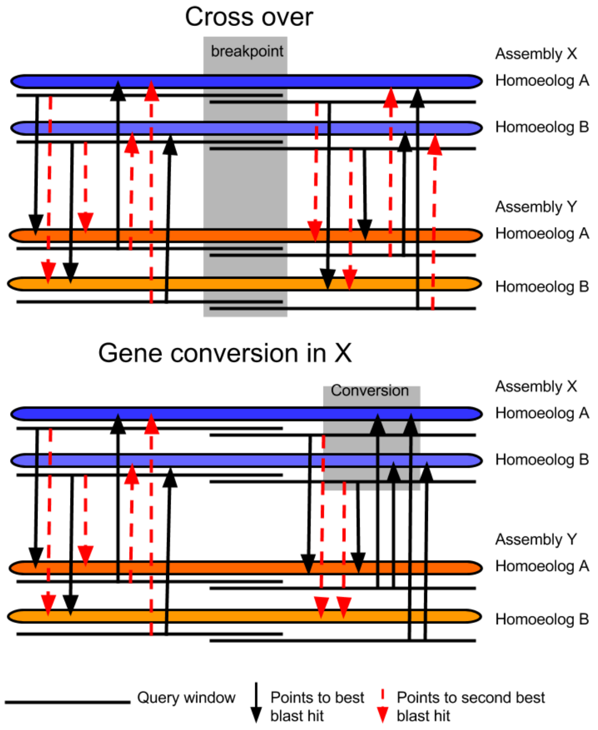

B

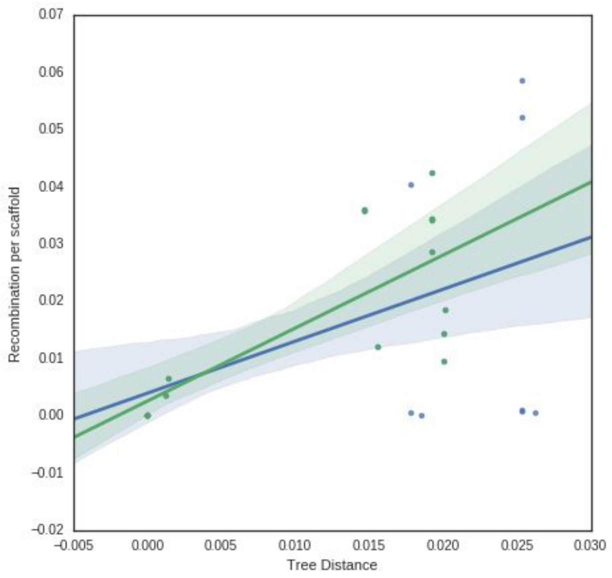

Figure S3: Detection of recombination events and distinction between gene conversion and recombination using BLAST (A). Correlation between MIG tree distances and gene conversion rates when including *M. floridensis* (blue, Pearson's r = 0.4) or excluding it (green, r = 0.74) (B).

Table S3: Recombination events per scaffold

	MareHarA	MfloSJF1	MincL27	MincW1	MjavVW4	MjavVW5
**MareHarA**	0.000000	0.052212	0.009484	0.034404	0.035968	0.011982
**MfloSJF1**	0.003972	0.000000	0.000000	0.003128	0.002062	0.000000
**MincW1**	0.028550	0.040255	0.003557	0.000000	0.034135	0.018472
**MjavVW4**	0.035750	0.058589	0.014226	0.042366	0.000000	0.006490

Exchange event counts between homologues (as in Figure S3 a) normalized by the number of the long scaffolds included in the analysis. The matrix is asymmetric: Y-axis samples served as a target in the blast analysis (Figure S3 a) and X-axis samples as subject.

3. Nuclear phylogenomic ML tree including the *M. incognita* isolate from (Abad et al. 2008).

Despite the wide geographic range of our *M. incognita* samples and the low genetic diversity they present, the Morelos strain from (Abad et al. 2008) seems to be an outgroup to the other *M. incognita* (Figure S3). Without access to the raw data and with the reported differences in sequencing and bioinformatics approaches it is difficult to explain these differences.

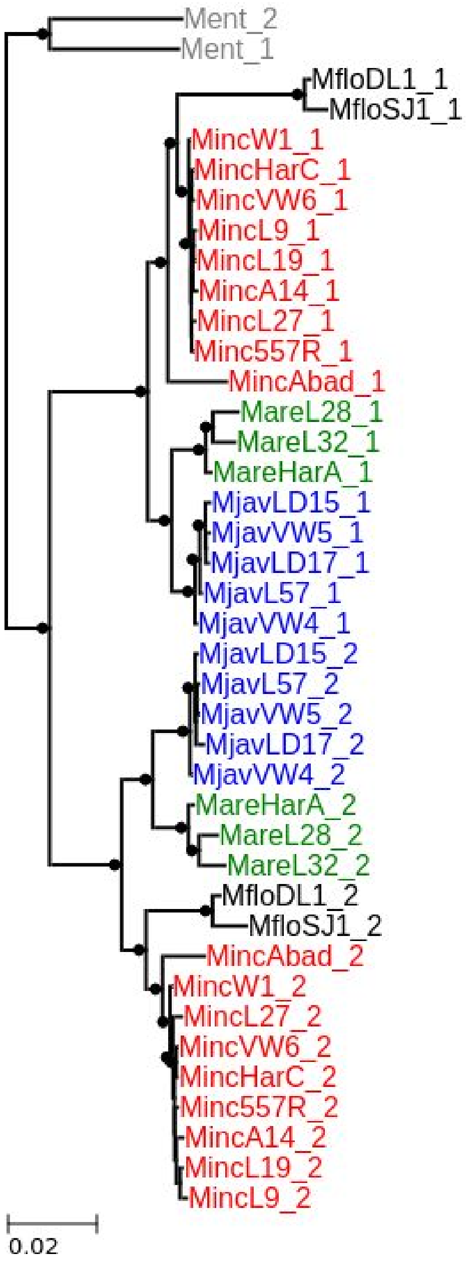

Figure S4: A maximum likelihood phylogenetic tree, of MIG and outgroup species. With the exception of the inclusion of the Morelos strain, it is identical to the tree in Figure 3.

4. Transposable elements

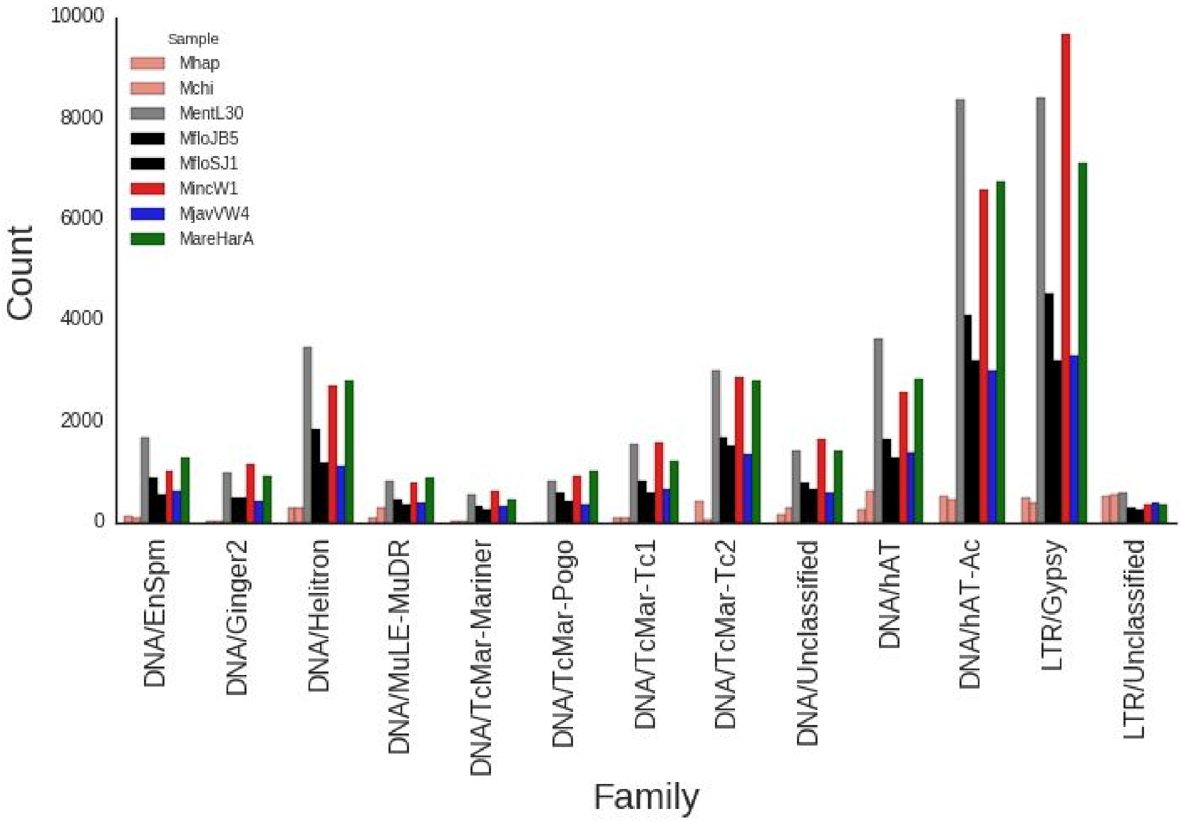

Figure S5: Counts of the 13 largest transposon families in the RKN genomes.

5. Revisit of previous orthology analyses

5.1 Gene trees of orthology clusters with three *Meloidogyne floridensis* copies

Supplementary data from (Lunt et al. 2014b) was downloaded from Figshare (http://dx.doi.org/10.6084/m9.figshare.978784). FastTree (Price, Dehal, and Arkin 2010) was used to reconstruct gene trees from the DNA sequence alignments of orthology clusters (OCs) with three copies for *M. floridensis*, as provided in the download. Among the 20 OCs, 4 supported the double hybrid hypothesis presented in (Lunt et al. 2014b), (e.g. Figure S6A), 7 appeared to represent two pooled OCs (e.g. Figure S6B), 7 contained *M. floridensis* inparalogues (e.g. Figure S6C), and two contained *M. floridensis* copies that had very little or no overlap.

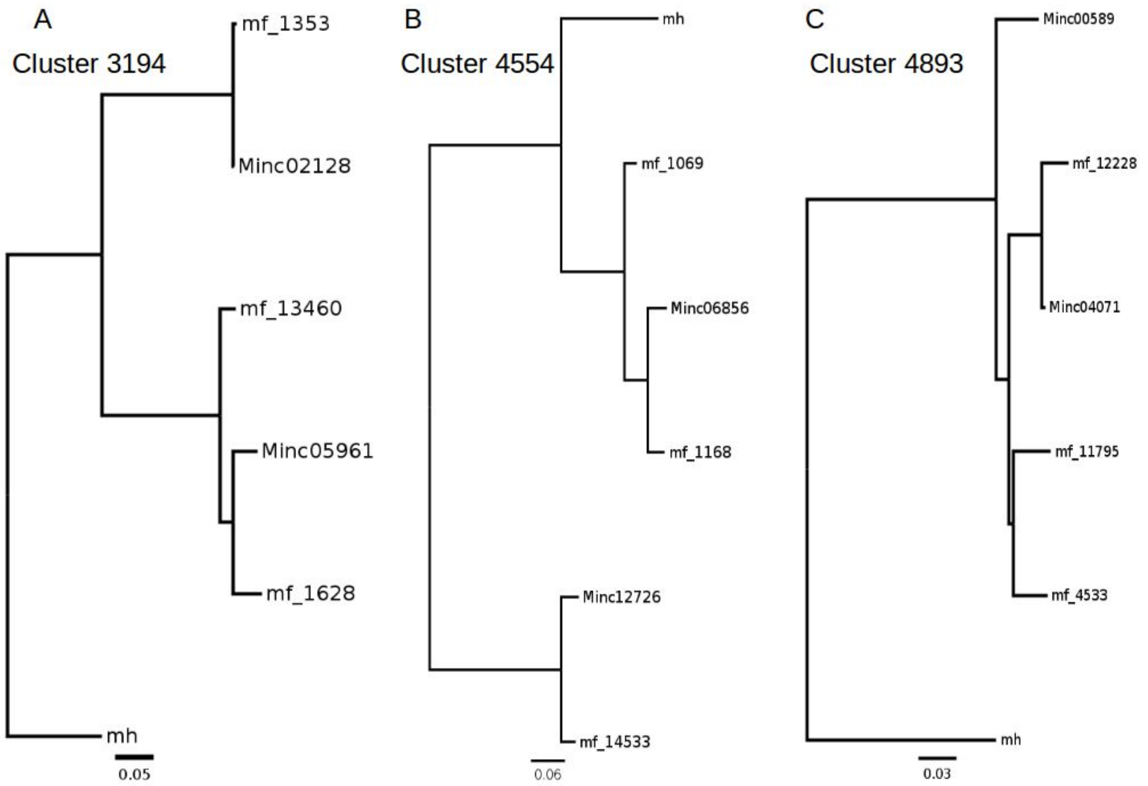

Figure S6: Phylogenetic patterns representing orthology clusters with three *M. floridensis* copies in the data of (Lunt et al. 2014b). Tree A represents clusters which support authentic three copies in *M. floridensis*, tree B represents clusters that appear to be a merge of two orthology groups, and tree C represents clusters with *M. floridensis* inparalogs. mf: *M. floridensis*, mh: *M. hapla*, Minc: *M. incognita*.

5.2 Gene trees of orthology clusters with three *Meloidogyne incognita* copies and two *M. floridensis* copies

Among 36 OCs with three *M. incognita* copies and two *M. floridensis* copies, 8 OCs support three genome copies in *M. incognita*, two of which are shared with *M. floridensis* (e.g. Figure S7A), 6 OCs recover other relationships (e.g. Figure S7B), 6 appear to be merged OCs (e.g. Figure S7C), 13 contain *M. incognita* inparalogs (e.g. Figure S7D), three OCs with "orthologs" that do not overlap.

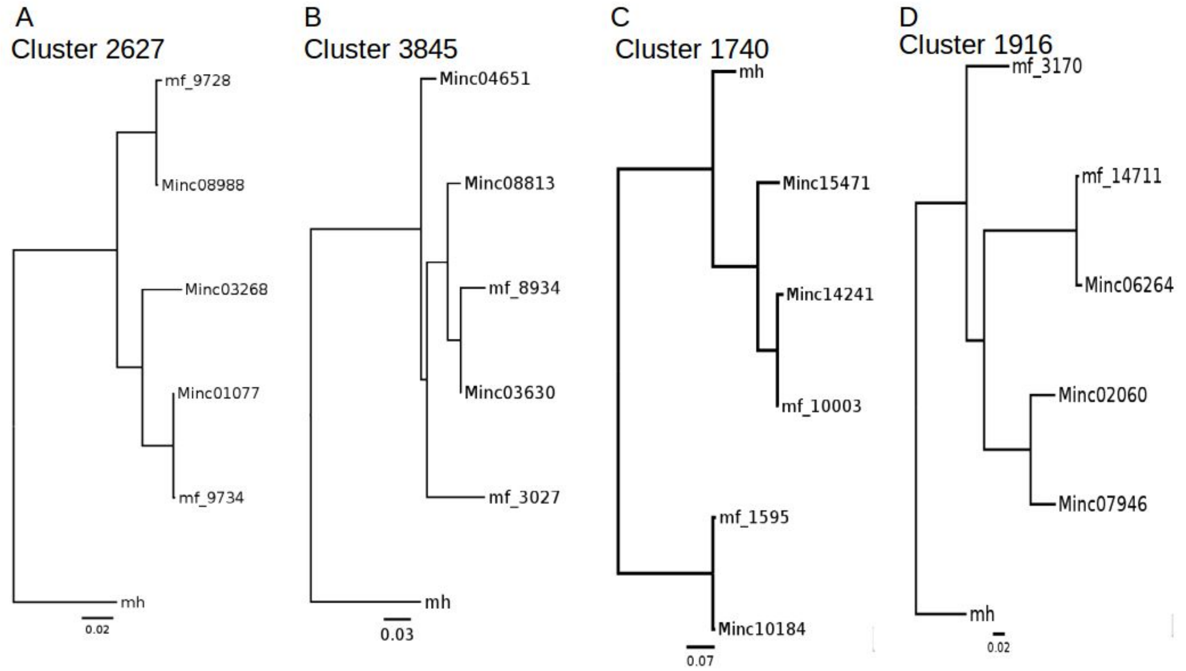

Figure S7: Phylogenetic patterns representing orthology clusters with three *M. incognita* copies in the data of (Lunt et al. 2014b). Tree A represents clusters which support authentic three copies in *M. incognita*, to of which are shared with *M. floridensis* tree B represents clusters with other relationships, tree C represents clusters that appear to be a merge of two orthology groups, and tree D represents clusters with *M. incognita* inparalogs. mf: *M. floridensis*, mh: *M. hapla*, Minc: *M. incognita*.

6. Phylogenetic analysis of mitochondrial genes

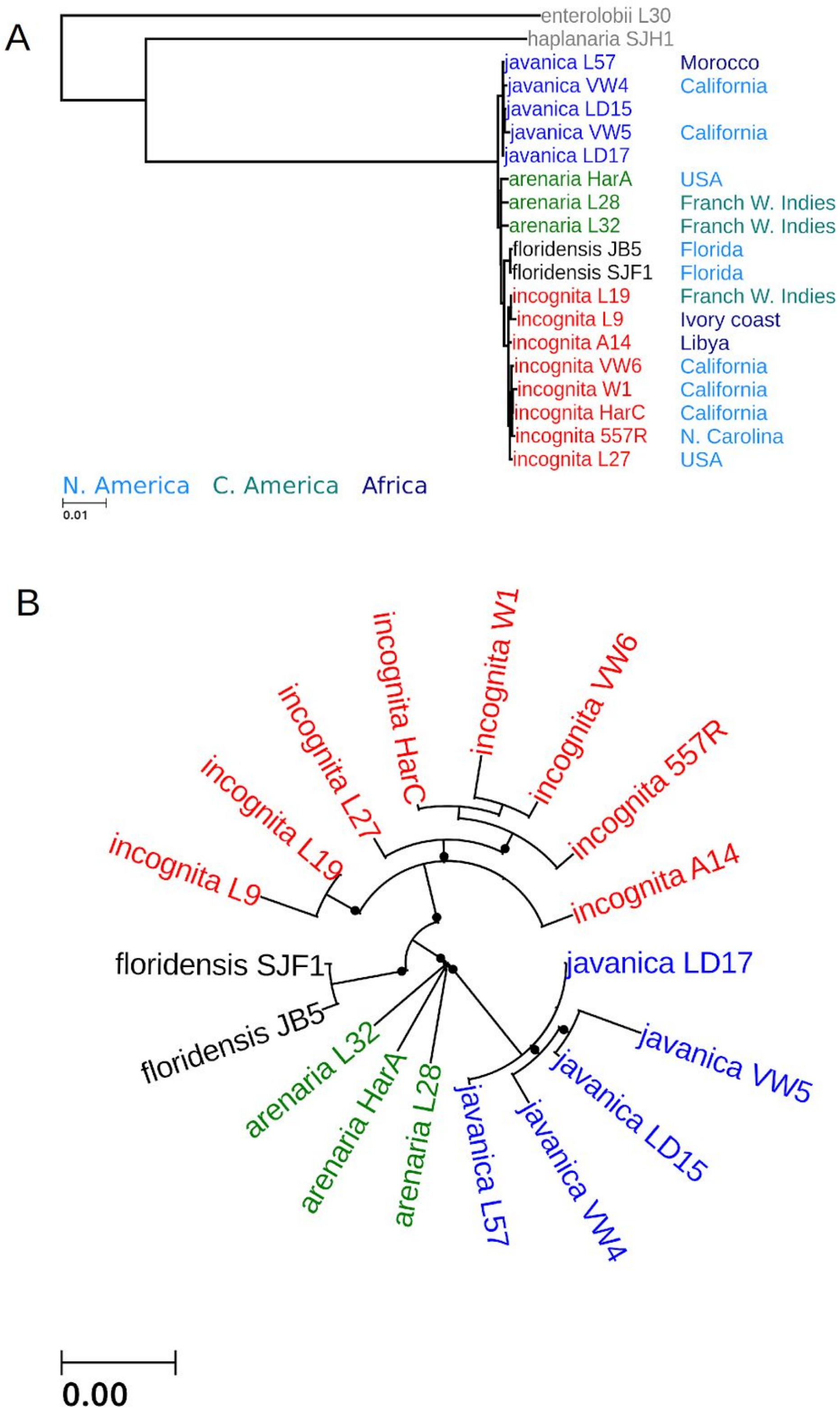

Figure S8: Rooted (A) and unrooted (B) maximum likelihood tree based on a concatenation of mitochondrial genes. Black bullets represent bootstrap percentage > 80 . The tree is in congruence with each of the homoeologue subtrees in the nuclear phylogenetic tree (Figure 3). The geographic origin of samples is indicated in A.

7. Phylogenetic analysis of mitochondrial genes

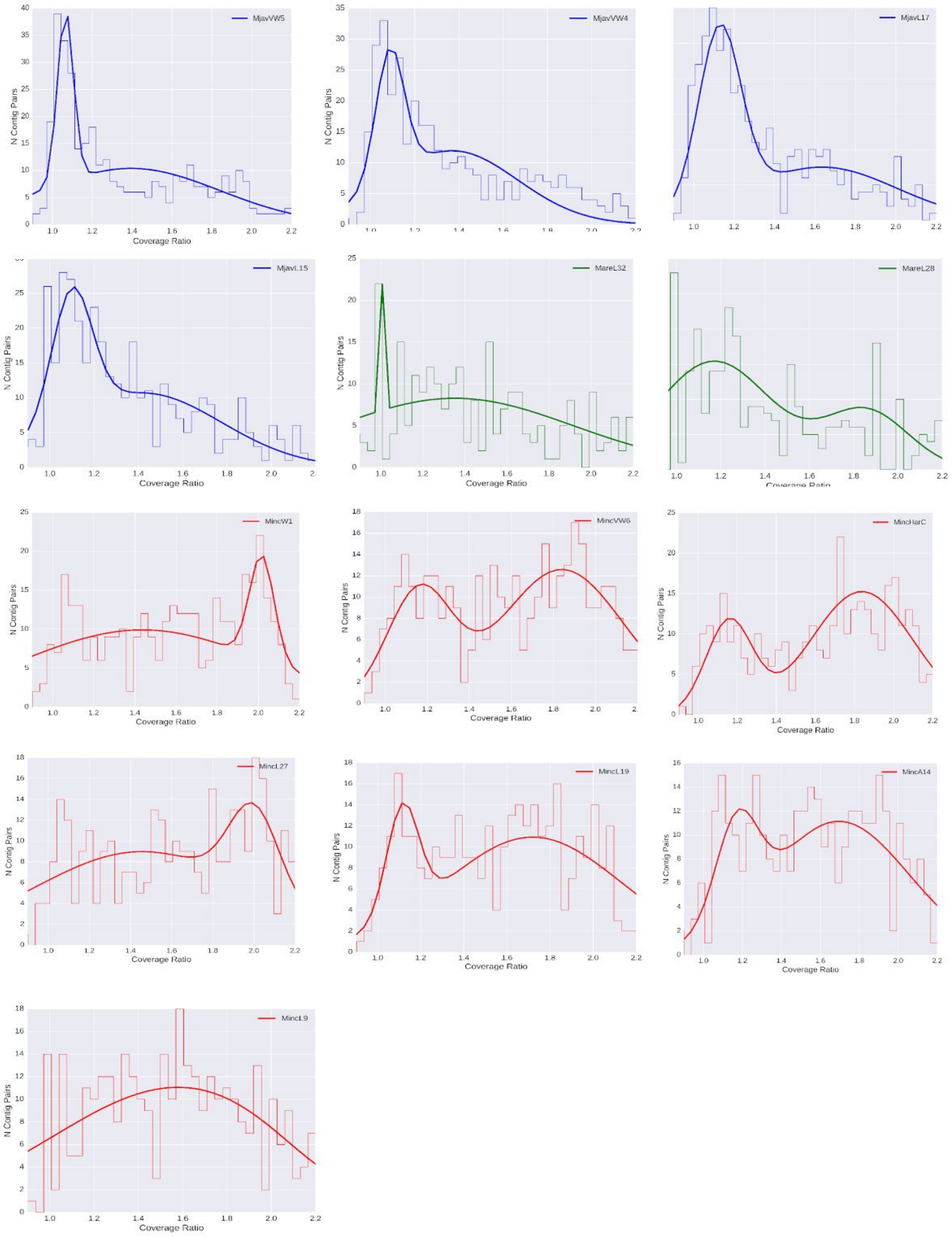

Figure S9: Median coverage ratio distribution per sample

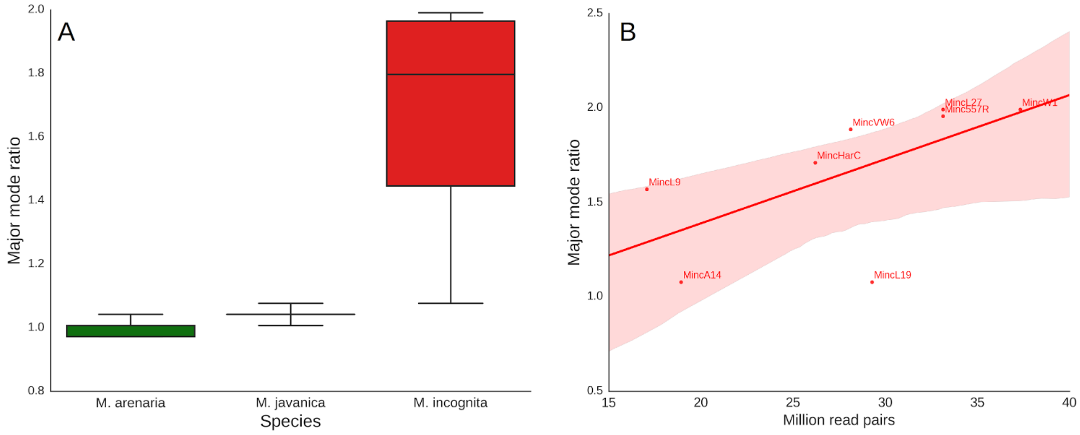

Figure S10: Coverage ratio at the major mode for each MIG apomict species. The major mode in M. incognita is around 2 (A), indicating that in this species a large proportion of the genome is triplicated, with two very similar copies and another ~3% divergent, or that there is more gene conversion between A1 and A2 in *M. incognita* than in other species. The large variance around the major mode value in M. incognita is an artifact of sequencing depth variation among M.incognita isolates, with deeply sequenced isolates demonstrating a clear signal for the large triplicated genome section (B).

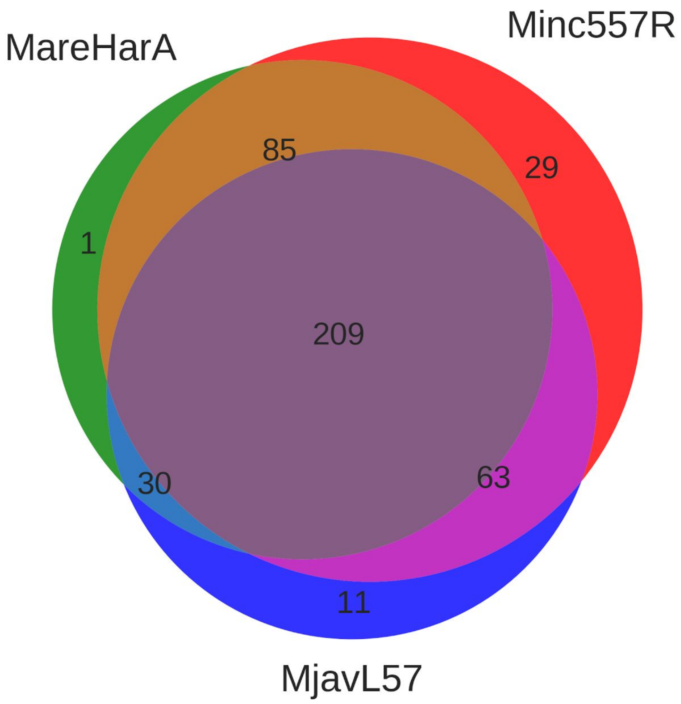

Figure S11: The number of shared contig pairs that represent A1,A2-B genome architecture in the three apomict MIG species.

8. Orthology Clusters

8.1 Shared orthology clusters

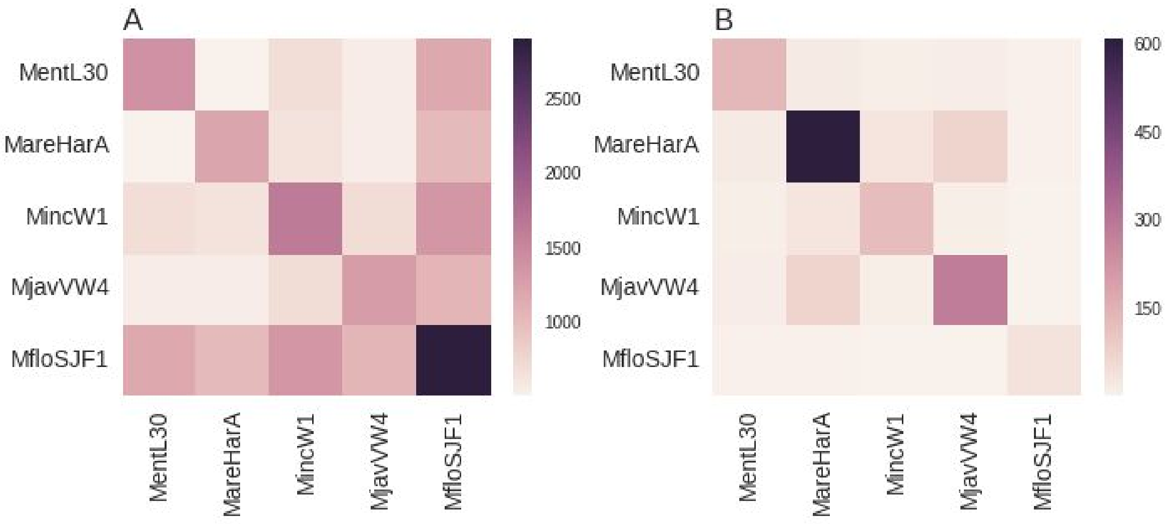

Figure S12: Shared orthology clusters with one (A) and three (B) copies between species

References

Abad, Pierre, Jérôme Gouzy, Jean-Marc Aury, Philippe Castagnone-Sereno, Etienne G. J. Danchin, Emeline Deleury, Laetitia Perfus-Barbeoch, et al. 2008. “Genome Sequence of the Metazoan Plant-Parasitic Nematode Meloidogyne Incognita.” *Nature Biotechnology* 26 (8): 909–15.

Adam, Mohamed A. M., Mark S. Phillips, and Vivian C. Blok. 2005. “Identification of *Meloidogyne* Spp. from North East Libya and Comparison of Their Inter- and Intra-Specific Genetic Variation Using RAPDs.” *Nematology: International Journal of Fundamental and Applied Nematological Research* 7 (4). Brill: 599–609.

Ferris, H., L. Zheng, and M. A. Walker. 2012. “Resistance of Grape Rootstocks to Plant-Parasitic Nematodes.” *Journal of Nematology* 44 (4): 377–86.

Gleason, Cynthia A., Qingli L. Liu, and Valerie M. Williamson. 2008. “Silencing a Candidate Nematode Effector Gene Corresponding to the Tomato Resistance Gene Mi-1 Leads to Acquisition of Virulence.” *Molecular Plant-Microbe Interactions: MPMI* 21 (5): 576–85.

Gross, Stephen M., and Valerie M. Williamson. 2011. “Tm1: A Mutator/foldback Transposable Element Family in Root-Knot Nematodes.” *PloS One* 6 (9): e24534.

Joseph, Soumi, Tesfamariam Mekete, Wiseborn B. Danquah, and Joseph Noling. 2016. “First Report of Meloidogyne Haplanaria Infecting Mi-Resistant Tomato Plants in Florida and Its Molecular Diagnosis Based on Mitochondrial Haplotype.” *Plant Disease* 100 (7): 1438–45.

Lunt, David H., Sujai Kumar, Georgios Koutsovoulos, and Mark L. Blaxter. 2014. “The Complex Hybrid Origins of the Root Knot Nematodes Revealed through Comparative Genomics.” *PeerJ* 2 (May). PeerJ Inc.: e356.

Price, Morgan N., Paramvir S. Dehal, and Adam P. Arkin. 2010. “FastTree 2–approximately Maximum-Likelihood Trees for Large Alignments.” *PloS One* 5 (3): e9490.

Wang, Congli, Steven Lower, Varghese P. Thomas, and Valerie M. Williamson. 2010. “Root-Knot Nematodes Exhibit Strain-Specific Clumping Behavior That Is Inherited as a Simple Genetic Trait.” *PloS One* 5 (12): e15148.

Yaghoobi, J., I. Kaloshian, Y. Wen, and V. M. Williamson. 1995. “Mapping a New Nematode Resistance Locus in Lycopersicon Peruvianum.” *TAG. Theoretical and Applied Genetics. Theoretische Und Angewandte Genetik* 91 (3): 457–64.
